# Screening and management of major bile leak after blunt liver trauma: a retrospective single center study

**DOI:** 10.1186/1757-7241-22-26

**Published:** 2014-04-15

**Authors:** Kuo-Ching Yuan, Yon-Cheong Wong, Chih-Yuan Fu, Chee-Jen Chang, Shih-Ching Kang, Yu-Pao Hsu

**Affiliations:** 1 Department of Trauma and Emergency Surgery, Chang Gung Memorial Hospital, Linkou, Chang Gung University, No.5, Fusing St, Gueishan Township Taoyuan County 333, Taiwan; 2 Division of Emergency and Critical Care Radiology, Department of medical Imaging and intervention, Chang-Gung Memorial Hospital, Chang-Gung University, Linkou, Taiwan; 3 Biostatistical Center for Clinical Research, Chang Gung, Memorial Hospital, Linkou, Taiwan

**Keywords:** Bile leak, Blunt liver injury, Liver injury, Blunt abdominal trauma, Endoscopic retrograde cholangiography, Jaundice, Trauma

## Abstract

**Background:**

Major bile leak after blunt liver trauma is rare but challenging. It usually requires endoscopic retrograde cholangiography (ERC) for management. However, there is still lack of specific indications. The aim of this study is to elucidate risk factors for major bile leak and indications for early ERC after blunt liver trauma.

**Methods:**

The trauma registry of a level I trauma center in Taiwan was queried, and all blunt liver trauma patients from June, 2008 to June, 2011 were selected for retrospective review. Data collected included demographic data, laboratory data, Injury Severity Score (ISS), liver injury grade and location, management of liver trauma, length of ICU, hospital stay and treatment result. ERC was used to confirm major bile leak.

**Results:**

288 blunt liver trauma patients were selected from 2,475 torso trauma patients. There were 214 (74.5%) male and 74 (25.7%) female patients. The mean ISS was 24.2. Most patients received conservative treatment. Transcatheter artery embolization (TAE) and operation were 15.6% and 10.8% respectively. Major bile leak occurred in 14 (4.9%) patients. Risk factors for bile leak include high-grade liver injury, centrally-located liver trauma and use of TAE. A bilirubin level greater than 43.6 μmol/L provides a sensitivity of 100% and specificity of 85.1% for predicting major bile leak.

**Conclusions:**

High injury grade; centrally-located liver trauma; and use of TAE are risk factors for major bile leak after blunt liver trauma. ERC should be arranged early if the patient has risk factors and their plasma bilirubin level is greater than 43.6μmol/L during admission.

## Background

Blunt liver trauma (BLT) can be severe and lethal. With the advancements in trauma treatment and interventional radiology, non-operative management (NOM) is now the standard of care for BLT if the hemodynamic is stable. The NOM has a high success rate as 85-95% [1,2]. With the high success rate, various late complications (infection, bleeding, biliary complications) have become emerging challenges. They are especially common in high grade (more than grade III) liver trauma [3,4]. These late complications usually hamper patient recovery seriously.

Although the incidence of major bile leak after BLT is low [1], it is crucial for patient recovery. Unlike iatrogenic bile duct injuries, bile duct injuries with bile leak after BLT are much more complex and subtle. The presentations are often nonspecific at first and an early precise diagnose is not easy. However, major bile leak after BLT carries high risk of infection; and it can significantly prolong hospital stay [5-7]. Therefore, timely recognition and management of bile leak is essential for patient recovery after BLT. This requires a high index of clinical suspicion and appropriately use of image studies [8,9]. At present, a follow-up imaging study after stable BLT is not a routine practice. Endoscopic retrograde cholangiography (ERC), which is very helpful for evaluation bile duct in iatrogenic bile duct injury, is rarely indicated early after BLT. Although ERC is the suggested modality for bile duct evaluation after liver trauma [10], it is mostly used when there is obvious jaundice. These patients who have jaundice often receive ERC late after BLT and resulted in prolong hospital stay and increased risk of infection. At present, there is still lack of an effective method to screen patients with potential major bile leak after BLT to receive early ERC.

The aim of this study was to review our experience of using ERC to detect major bile leak after BLT. By analyzing the treatment result of our patients, we try to elucidate risk factors and to propose indications for early ERC after BLT in order to reduce the hospital stay.

## Materials and methods

### Study design

This was a retrospective chart review study. The trauma registry database of Chang Gung Memorial Hospital- Linkou, a level I trauma center in Taiwan, was reviewed to identify all torso trauma patients from June 2008 to June 2011. Torso trauma including liver trauma were selected for further analysis. Patients who sustained penetrating injuries were excluded. Charts and other medical records of all the included patients were reviewed.

### Ethical approval

This study was approved by the Institutional Review Board of Chang Gung Memorial Hospital, Linkou.

### Initial management

In general, these patients were severely injured, and they underwent managements according to our protocol based on the Advanced Trauma Life Support (ATLS). Torso trauma patient who had stable hemodynamic status or had a good response to fluid resuscitation received a Computed Tomography (CT) scan with intravenous (IV) contrast when abdominal or pelvic injuries were suspected. CT scans were performed using a 64-multidetector CT machine (LightSpeed QX/i Scanner, General Electric Medical Systems, Milwaukee, WI, USA), which was located adjacent to the surgical resuscitation room. IV contrast agent was routinely administrated unless there was contraindication. An uniphasic injection of 100–120 ml of contrast agent was given to the patient at a rate of 1-3 ml/s, and images of 5–10 mm collimation and 5–8 mm reconstruction intervals were obtained 60–70 seconds after the administration of intravenous contrast medium administration.

Liver injury was graded according to American Association for the Surgery of Trauma (AAST) Organ Injury Scale of liver by the results of CT scan. Liver trauma was managed in a multidisciplinary fashion, including surgery, transcatheter artery embolization (TAE), or non-operative management (NOM) with close monitoring and blood transfusion only. TAE was used for primary hemostasis or as an adjunctive for surgery. All these patients were admitted to the Trauma Intensive Care Unit (TICU) for close monitoring and further care after initial management.

### Bile leak evaluation

In our hospital, we do not routinely perform follow-up imaging for BLT patients unless there are clinical presentations suggestive of complications. Indications for follow-up imaging studies after BLT include fever, persistent abdominal pain or fullness, gross jaundice, or abnormal content in surgical drainage device. If abnormal intra-abdominal fluid collection is detected in CT scan or abdominal sonography, further management is arranged according to clinician’s expertise and decision. Some patients received percutaneous drainage, but some received conservative treatment only. If bile stain was noted in the percutaneous drainage device, bile duct injury was considered highly possible and ERC was indicated. If the patients received laparotomy for liver trauma initially and had high bilirubin level in drainage content or grossly bile stained content in postoperative drainage device, they were also indicated for ERC.

A major bile leak was defined as having definite contrast leaking from bile duct on ERC. If bile duct injuries were confirmed on ERC, a method for bile flow diversion will be applied using stent or nasobiliary catheter. Successful treatment of bile duct injury was defined as resolution of clinical symptoms as well as all of the followings: resolution of bile leak on the follow-up ERC, removal of both bile diverting device and percutaneous drainage catheter smoothly.

### Data collection

Data collection includes demographic data, trauma mechanism, Injury Severity Score (ISS), length of mechanical ventilator (MV duration) use, length of TICU stay (ICU duration), length of hospital stay (LOHS) and the initial presentations in triage. The highest serum level of AST, ALT and bilirubin during admission was also collected.

### Injury location definition

**Figure 1:**
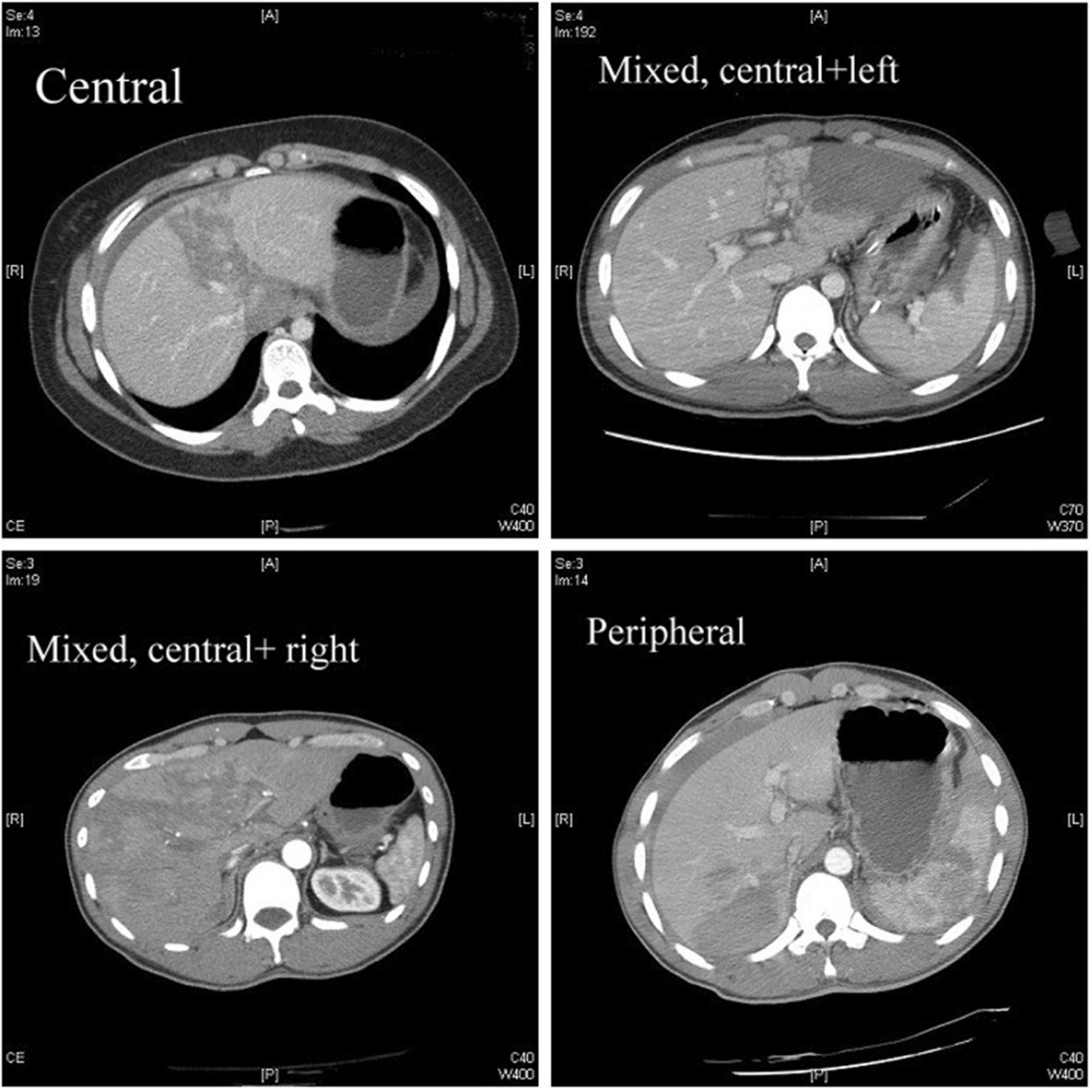
Different locations of liver injury.

In addition to the AAST grading for liver injury, we further divided liver injuries into central (involving segments 4, 5, or 8), peripheral (involving segments 2, 3, 6, or 7), or mixed (involving both central and peripheral segments) based on abdominal CT (Figure 1).

### Statistics

Numerical data are expressed as the mean ± standard deviation, and SPSS version 16 (SPSS, Chicago, IL, USA) was used for analyses. A p value of less than 0.05 was considered statistically significant. Continuous numerical variables were analyzed with a two-sample t-test or one-way ANOVA. Categorical variables were analyzed with the Chi-square test or Fischer’s exact test. A receiver operating characteristic (ROC) curve was produced and the area under the curve was calculated for the model to predict major bile leak with different bilirubin levels.

## Results

### Study population

**Figure 2:**
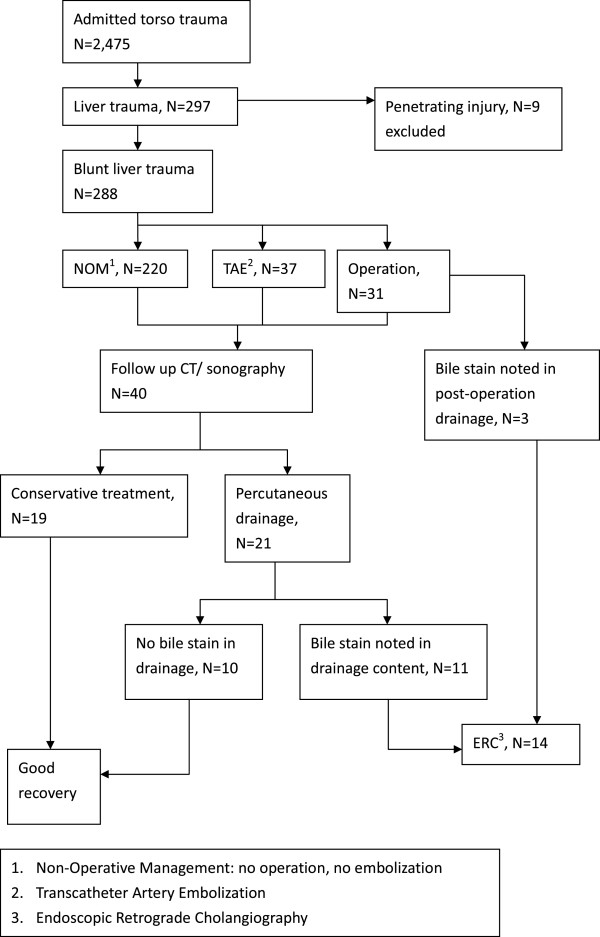
Flowchart of querying and management of patients in the study.

There were 2,475 torso trauma patients admitted during study period, and 297 (12%) patients presented with liver trauma (Figure 2). Nine patients were excluded because they sustained penetrating injuries. Therefore, 288 blunt liver trauma patients were eligible for our study (Table 1). There were 214 male and 74 female patients, and the mean age was 34.5 years. The mean ISS was 24.2 and the most common trauma mechanism was motorbike accident. Grade III liver injury was the most common injury grading (31.3%), and 60.4% patients had high grade injuries (grade III -V). Regarding to the injury location, the peripheral type was the most common (54.2%).

**Table 1:** Demographic data of all blunt liver injury patients

**Case number (N)**	**288**
Age (year)	34.5 ± 15.2
ISS	24.2 ± 11.8
SBP in ED (mm/Hg)	116.9 ± 34.5
Sex	
Female	74 (25.7%)
Male	214 (74.3%)
Trauma mechanism	
Motor Bike Accident	198 (68.8%)
Fall	30 (10.4%)
Fight	4 (1.4%)
Heavy compression	1 (0.3%)
Pedestrian Accident	17 (5.9%)
Motor Vehicle Accident	38 (13.2%)
Liver injury grade	
1	40 (13.9%)
2	74 (25.7%)
3	90 (31.3%)
4	79 (27.4%)
5	5 (1.7%)
Liver injury location	
Central	112 (38.9%)
Mixed	20 (6.9%)
Peripheral	156 (54.2%)
Treatment	
No intervention	219 (76%)
Embolization	45 (15.6%)
Operation	31 (10.8%)
Result	
Bile leaks	14 (4.9%)
Expired	23 (8%)

*SBP* systolic blood pressure.

*ED* Emergency Department.

### Initial management

Most of these patients (76%) received NOM without operation nor TAE. 15.6% patients underwent TAE, and 10.8% patients underwent an operation respectively for liver trauma management. Twenty three patients died after treatment and the mortality rate was 8%.

### Bile leak evaluation

After initial management, 40 patients (13.8%) received follow-up imaging studies (CT scan or sonography) at different time intervals during admission due to various clinical indications such as prolong fever, fatigue, abdominal fullness or poor appetite. These 40 patients all had intra-abdominal fluid collections (intrahepatic or intraperitoneal) detected on CT or sonography. Nineteen of them received only conservative treatment without further intervention and all recovered well. The rest 21 patients received percutaneous drainage and 11 of them had bile stained drainage content. The eleven patients received ERC later and they all had major bile leak on ERC. Among these 11 patients, four received NOM and seven received TAE as initial management for BLT. Their conditions were stabilized after TICU admission and the major bile leak was confirmed late during ward admission. Another three patients who also had major bile leak on ERC underwent an operation for BLT initially. One patient had the operation at other hospital immediately after BLT and was transferred to our hospital for further treatment. The bilirubin level in his surgical drainage device was 889.2 μmol/L and major bile leak was highly suspected. Two patients had operation at our hospital due to BLT. They both had suspicious bilious drainage several days after operation with abdominal fullness. ERC was therefore used to exclude a possible major bile leak.

### Management for bile leak

All these fourteen patients who received ERC had major bile leak confirmed by ERC, so the incidence of major bile leak after BLT in our study was 4.9%. During ERC, sphincterotomy was performed and bile flow was either stented or nasobiliary drained for all patients but 2 failed. For the two failed patients, one patient received conservative treatment only and recovered. The other patient underwent liver lobectomy soon after ERC and also recovered uneventfully. A follow-up ERC was arranged approximately 1 month later for the evaluation of bile duct recovery or stent exchange. All patients recovered smoothly from bile leaks after 2-3 times of ERC procedures with stent exchange.

### Treatment result and analysis

**Figure 3:**
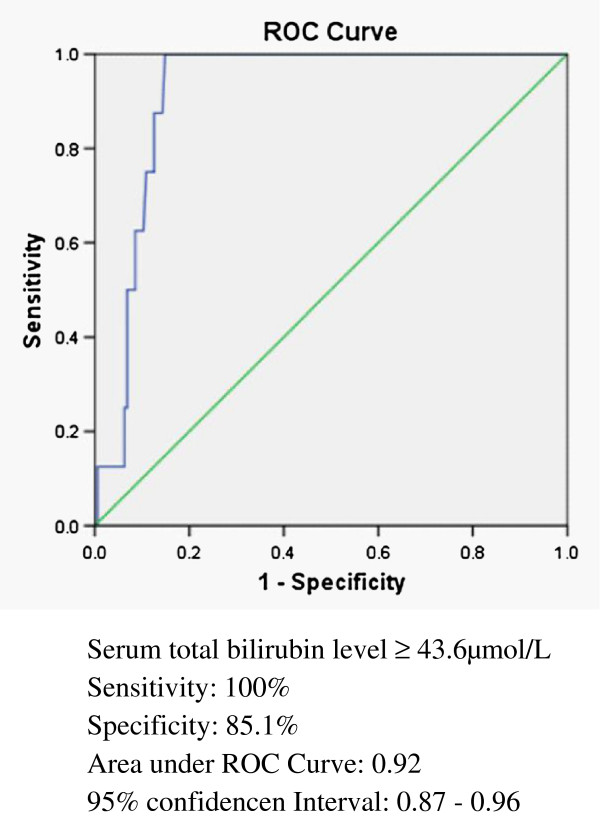
Receiver operating characteristic (ROC) curve for plasma bilirubin level and the subsequent major bile leak after blunt liver trauma.

There was no mortality among the patients with major bile leak. After treatment, however, the length of hospital stay was significant longer for the major bile leak patients (Table 2). In analysis, we found high grade liver injuries, location of injury, liver enzyme (ALT) and total bilirubin level were significant for bile leak patients. With respect to management of BLT, patients who received NOM were less likely to have major bile leak then operation or TAE. In fact, TAE was significant for major bile leak in our study (Table 3). After using a ROC curve for analysis, we noticed that using a serum bilirubin level 43.6μmol/L can provide a sensitivity of 100% and specificity of 85.1% for predicting major bile duct injury (Figure 3).

**Table 2:** Analysis between bile leak patients and No-Bile-Leak patients

	**Bile Leak**	**No-Bile-Leak**	***p***
**Patient number**	14 (4.9%)	274 (95.1%)	
**Age (years)**	32+ 19.4	35.6 ± 15.1	*0.72*
**ISS**	27.9 ± 7.6	24 ± 11.9	*0.37*
**SBP in ED (mmHg)**	112.8 ± 42.4	117 ± 34.3	*0.73*
**Sex**			*0.97*
**Male**	10 (71.4%)	204 (74.5%)	
**Female**	4 (28.6%)	70 (25.5%)	
**Trauma mechanism**			*0.85*
**Motor Bike Accident**	12 (85.7%)	186 (67.9%)	
**Fall**	0	30 (10.9%)	
**Fight**	0	4 (1.5%)	
**Heavy compression**	0	1 (0.4%)	
**Pedestrian Accident**	0	17 (6.2%)	
**Motor Vehicle Accident**	2 (14.3%)	36 (13.1%)	
**Liver Injury Grade**			0.007
**1 & 2**	0	114 (41.6%)	
**3**	4 (28.6%)	86 (31.4%)	
**4**	9 (64.3%)	70 (25.5%)	
**5**	1 (7.1%)	4 (1.5%)	
**Liver injury location**			<0.01
**Central**	9 (64.3%)	103 (37.6%)	
**Mixed**	5 (35.7%)	15 (5.5%)	
**Peripheral**	0	156 (56.9%)	
**Laboratory data**			
**AST (U/L)**	1171 ± 1352.6	658.9 ± 1595.5	0.07
**ALT (U/L)**	1466.1 ± 1071	406.3 ± 500.7	<0.01
**Total Bilirubin ** **(μmol/L)**	119.7 ± 128.3	35.9 ± 54.7	<0.01
**Admission course**			
**TICU duration (Day)**	9.2 ± 8	5.2 ± 6	0.07
**MV duration (Day)**	5 ± 6.3	2.6 ± 5.3	0.21
**LOHS (Day)**	40 ± 39.7	16.3 ± 16.1	<0.01
**Outcome**			0.5
**Survived**	14 (100%)	251 (91.6%)	
**Expired**	0	23 (8.4%)	

*SBP* systolic blood pressure.

*MV* mechanical ventilator.

**Table 3:** Management of blunt liver trauma and bile leak

	**Bile Leak (N = 14)**	**No Bile Leak (N = 274)**	***P***	**Odds Ratio**	**95% Confidence Interval**
Intervention or not			0.002	3.48	2.17- 5.59
Intervention (OP or TAE)	10 (14.5%)	59 (85.5%)			
No intervention	4 (1.8%)	215 (98.2%)			
TAE			0.02	3.6	1.67- 7.74
E	7 (15.6%)	38 (84.4%)			
NE	7 (2.9%)	236 (97.1%)			
Operation			0.21	2.42	0.69- 8.56
Operation	3 (9.7%)	28 (90.3%)			
No operation	11 (4.3%)	246 (95.7%)			

*OP* operation.

*TAE* transcatheter artery embolization.

*E* embolization.

*NE* no embolization.

## Discussion

Bile leak is one of the major complications after all kinds of liver trauma and the reported incidence ranges from 0.5 to 21% [1,2]. However, the possibility of bile duct injury does not reduce the safety and application of non-operative management for blunt liver trauma [11]. Most of the bile leaks after blunt liver trauma are minor and recovered well after conservative treatment [12]. Major bile leak, however, can seriously hamper patient recovery and is an emerging issue in treatment for blunt liver trauma [3,4,9,13]. The key for treatment of major bile leak is timely diagnosis and effective intervention. Early treatment with bile flow diversion can prevent the development of further complications such as biloma, infection or even intraabdominal sepsis. Therefore, it is helpful to recognize patients who possess high risk for major bile leak in order to provide managements early.

The injury grade and location of blunt liver trauma are significant factors for later major bile leak in this study (Table 2). In the literature, blood transfusion amount within the first 24 hours after trauma and high grade liver injury has been proposed risk factors for complications after liver trauma [3]. Wahl et al. discussed bile duct injuries after blunt liver injury and found that patients sustained high injury grade (> grade IV) were more likely to have bile leaks [2]. In our study, high injury grade is also a significant factor for major bile leak. No bile leak happened in grade I or II liver trauma; and the incidence of major bile leak was highest in grade IV (Table 2). Injury location was another significant factor for major bile leak in our study, and this was not discussed before. Centrally located liver injuries were more likely to develop major bile leak than peripherally located ones (Table 2). We considered injury location as a reasonable and relevant risk factor. Because the intrahepatic bile ducts distribute in a confluent manner and the main bile ducts are located more centrally. Main bile duct is more likely to be injured if the blunt liver trauma involves the central parts. When the main bile duct is injured, it is more difficult to recover then the peripheral located small bile ducts, and major bile leak is therefore more likely to happen.

The initial management method for blunt liver trauma is also relevant for major bile leak in our study (Table 3). Patients who received TAE for initially have significant higher incidence of major bile leak in our study than patients who received surgery or NOM. A similar result had been reported. In a study regarding complications after liver injury; the incidence of a bile leak in patients who received TAE is 41.2%; whereas it is only 19.2% for those who received operation; and 1.5% for those who were only observed [2]. There was no literature thoroughly addressing about this. Since major hepatic necrosis and gallbladder ischemia had been noted as the major complications after hepatic artery embolization after major liver trauma [14,15]; we therefore presumed that a potential contributor to this result is impaired perfusion of the bile duct epithelium after TAE. Because of the damage control concept in trauma management, non-selective TAE is often applied in liver trauma. Therefore, the adverse effects of tissue ischemia after TAE are more extensive and profound. For patients who received TAE after blunt liver trauma, the bile duct epithelium sustained dual injuries: the mechanical tissue destruction during trauma and the ischemia injury after TAE. A poor healing process of the injured bile duct is highly possible and the bile leaks occurred subsequently. However, this presumption needs further verification.

For those who possess high risk for major bile leak, further evaluation is necessary. These patients usually have many painful injuries, hemoperitoneum, or other non-specific abdominal complaints that hamper the precise evaluation of bile duct at first. However, those non-specific abdominal complaints can be regarded as a first presentations for bile duct injuries if it persists. In our study, patients without these abdominal complaints later all recovered well. For patients who have abdominal symptoms, further ancillary studies are necessary. There are several modalities suggested for bile flow or bile duct integrity evaluation, including radionuclide scan [2,16], magnetic resonance imaging (MRI) [17,18], CT scan, or sonography. ERC is very accurate for bile duct evaluation but is too invasive to be used as the routine evaluation modality. CT scan or sonography was the first line examination in this study. Intraperitoneal or intrahepatic fluid accumulation is often the most common finding for these patients, however, furthur intervention is not always mandatory [19]. In our study, 40 patients had loculated intra-abdominal fluid but nineteen (47.5%) recovered well after conservative treatment. Percutaneous drainage for the fluid is usually the next step if clinical recovery is not satisfactory [20]. Adequate perihepatic drainage has been suggested to prevent repeat laparotomy for bile peritonitis after liver trauma [11]. In our study, twenty-one patients underwent percutaneous drainage and 10 of them recovered well after percutaneous drainage. By our study result, as much as 72.5% (19 + 10 of 40) of patients with intraabdominal fluid after blunt liver trauma can be managed with conservative treatment with or without percutaneous drainage and only 27.5% need further ERC due to major bile leak.

The most widely accepted indication for ERC after blunt liver trauma is jaundice after trauma. However, jaundice after liver trauma is very complex and the causes are multiple. It includes resolving hematoma after trauma, transfusion related hyperbilirubinemia, progressing of infection, hepatic dysfunction after injury, or bile duct injury [21]. Most of the jaundice after blunt liver trauma needs only conservative treatment; except bile duct injury related jaundice. Nevertheless, we still lack a reference level using plasma bilirubin to differentiate patient who needs only conservative treatment or who is more likely to have major bile leak. Watchful waiting is a common practice since most of the jaundice will recover after conservative treatment. But this practice often results in delayed management and prolonged hospitalization. In our study, the average interval between liver trauma and the first ERC was 25.8 days and the average length of hospital stay was significantly longer for those who had major bile leak (Table 2). This is because not until more clinical presentations suggestive of bile leaks happened that we will arrange ERC. The length of hospital stay could have been reduced if ERC had been performed early. There was study suggesting that ERC be performed in conjunction with initial CT scan [4]. However, this timing for ERC is too close to the liver trauma and may result in overdiagnosis of minor bile duct injuries that will resolve spontaneously. It is also not so feasible to arrange ERC for all blunt liver trauma patients at the initial stage as a routine practice because of the invasiveness of this procedure and the unstable condition of the patient.

**Figure 4:**
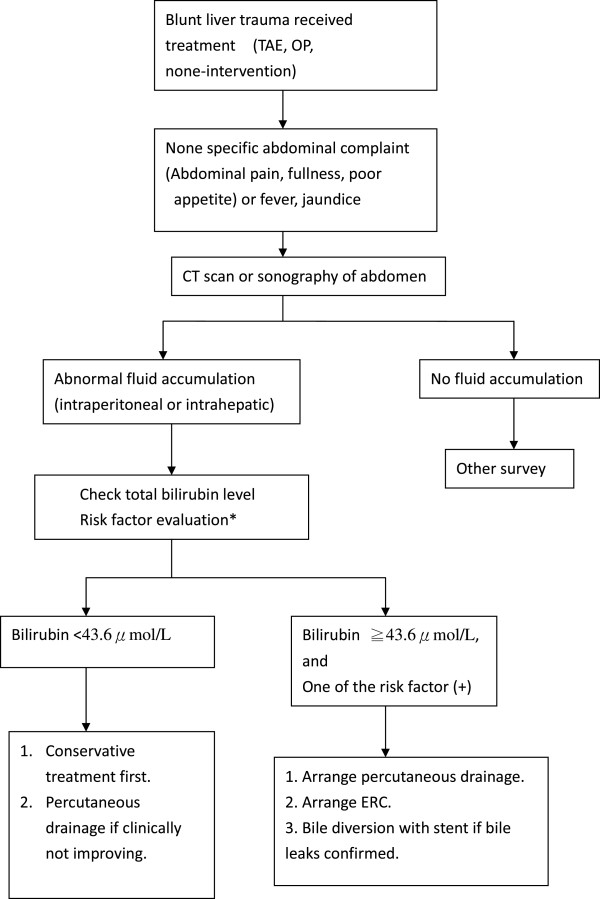
Flowchart for major bile leak screening after blunt liver trauma.

In our analysis, blunt liver trauma patients with major bile leak had higher plasma bilirubin level then those who did not; and this different was indeed significant (Table 2). After using a receiver operating characteristic (ROC) curve for analysis, we noticed that using a cut off value of 43.6μmol/L for plasma bilirubin can provide a sensitivity of 100% and specificity of 85.1% to predict major bile duct injury (Figure 3). This was not been discussed before in literature. Although our study was retrospective and the sample size was small, useful data for creating an algorithm for evaluation of major bile leak after blunt liver trauma had emerged (Figure 4). Patients without any abdominal complaints after blunt liver trauma need no further image studies. Patients who have any non-specific abdominal complaints after BLT should receive further studies, including CT scan or sonography and laboratory follow up. If abnormal intraabdominal fluid accumulation is detected, the plasma bilirubin level is crucial. If the bilirubin level is less than 43.6μmol/L, conservative treatment is suggested. If the bilirubin level is greater than 43.6μmol/L or patient has one or more of the risk factors (high grade liver injury, centrally located injury, receive TAE of liver); percutaneous drainage along with ERC should be arranged promptly. A bile diversion method should be applied if ERC confirmed major bile leak. Following this algorithm, major bile leak can be detected and managed earlier than before; and the length of hospital stay should be reduced.

There are some limitations of our study inherent to its retrospective nature. Because bile duct injury is rare, our patient numbers are relatively small. There was no established management protocol for this complication; our management was at the discretion of the each trauma surgeon’s expertise and experience. Therefore, the long length of hospital stays for these patients is partially biased by individual trauma surgeons’ principles. Further prospectively designed studies should be conducted to verify our proposed algorithm and to evaluate its effect on hospital stay.

## Conclusions

In conclusion, major bile leak after blunt liver trauma is uncommon, and its incidence is approximately less than 5%. In addition to a high grade injury, centrally-located liver injuries and initial TAE are also significant risk factors for major bile duct injury. For blunt liver trauma patients who developed abnormal fluid collections and some non-specific complaints; along with a bilirubin higher than 43.6μmol/L or having any one of the proposed risk factors, ERC should be considered indicated due to high possibility of major bile leak and to provide early intervention.

## Competing interest

All authors declared no conflict of interest and disclosed no external funding in this study.

## Authors’ contributions

KC Yuan started out the primary study design and organized the work in data collection. CY FU and SC Kang participated in data collection. YC Wong participated in reviewing of image and collection of data about image. CJ Chang participated in and performed the statistical analysis. KC Yuan drafted the manuscript. YP Hsu conceived of the study, and participated in its design and coordination and helped to draft the manuscript. All authors read and approved the final manuscript.
